# The Effect of a Stroop-like Task on Postural Control in Dyslexic Children

**DOI:** 10.1371/journal.pone.0077920

**Published:** 2013-10-28

**Authors:** Maria Pia Bucci, Emmanuel Bui-Quoc, Christophe-Loic Gerard

**Affiliations:** 1 UMR 676, Hôpital Robert Debré, 75019, Paris, France; 2 Service d’Ophtalmologie, Hôpital Robert Debré. Paris, France; 3 Service de Psychopathologie de l’enfant et de l’adolescent. Hôpital Robert Debré, Paris, France; The Ohio State University, Center for Cognitive and Brain Sciences, Center for Cognitive and Behavioral Brain Imaging, United States of America

## Abstract

The influence of a secondary task on concurrent postural control was explored in twenty-one dyslexic children (mean age: 10.4±0.3 years). Data were compared with twenty age-matched non-dyslexic children. As a secondary task, a modified Stroop test was used, in which words were replaced with pictures of fruits. The postural control of children was recorded in standard Romberg condition as the children were asked to name the colour of fruits appearing consecutively on a computer screen. Two conditions were tested: a congruent condition, in which the fruit was drawn in its natural ripe colour, and a non-congruent colour condition (NC), in which the fruit was drawn in three abnormal colours. A fixating condition was used as baseline. We analyzed the surface, length and mean speed of the center of pressure and measured the number of correct responses in the Stroop-like tasks. Dyslexic children were seen to be significantly more unstable than non-dyslexic ones. For both groups of children, the secondary task significantly increased postural instability in comparison with the fixating condition. The number of correct responses in the modified Stroop task was significantly higher in the non-dyslexic than in the dyslexic group. The postural instability observed in dyslexic children is in line with the cerebellar hypothesis and supports the idea of a deficit in automatic performance in such children. Furthermore, in accordance with cross domain competition model, our findings show that attentional resources are used to a greater extent by the secondary task than in controlling body stability.

## Introduction

Postural control involves the integration of visual, vestibular and proprioceptive inputs in order to produce correct motor commands to control the body’s position in space [1]. In everyday life, attentional resources used to control posture are frequently shared so as to perform other tasks simultaneously; thus postural stability is naturally part of a dual task.

Kerr et al. [2] were the first to show that postural control in young adults is attention-dependent and that postural stability is affected by a secondary task. Blanchard et al. [3] have explored the effects of a secondary task on balance in eight- to ten-year-old children and reported an improvement in postural stability, i.e. smaller sway variability, when children were performing a task such as counting backwards or reading a sentence, compared to that recorded when they were looking at an image. Schmid et al. [4] using a similar task (mentally counting backwards), showed a strong perturbation of postural stability in nine-year-old children. Similarly Olivier et al. [5] reported an increase in instability in seven-year-old children when they were asked to perform a modified Stroop task. All these findings have shown a significant interaction between attentional processes and balance performance. According to Olivier et al. [5] two independent attentional mechanisms could exist during a dual task; one instrumental in controlling posture and the other responsible for the secondary task. These two mechanisms could interfere with each other depending on the difficulty of the dual – cognitive and postural – task.

Dyslexia is a neurobiological disorder characterized by a difficulty in reading acquisition despite adequate intelligence, conventional education and motivation [6]. Frank and Levinson [7] were the first to report subjectively that dyslexic children showed neurological signs of cerebellar-vestibular deficiency (i.e., positive Romberg test, difficulty in tandem walking, articulatory speech disorders, hypotonia, and several dysmetric deficits). The cerebellar deficit hypothesis was confirmed by Nicolson and Fawcett [8] showing deficit in balance and motor coordination in a population of dyslexic children; their postural stability was affected by a secondary task, shifting attention away from the primary postural one. These authors suggested that dyslexics need to invest more attentional resources than non-dyslexics to control their balance when two tasks are performed simultaneously. Several subsequent studies showed that dyslexic subjects had poor motor performance during a postural control single task as well as in one associated with another task. Moe-Nilssen et al. [9] showed impairment of both balance and gait capabilities in dyslexic children compared to non dyslexic children of similar age (10–12 years old); Brookes et al. [10] also reported on poor postural control in children and adults with dyslexia. Vieira et al. [11] showed that during a dual task (reading single words) the postural stability of dyslexic children decreased significantly. A subsequent study of the same group [12] showed that a vibration of the ankle muscles impaired stability more strongly in dyslexic than in non-dyslexic children, independently of the attentional task. Moreover, in the condition without vibration, the attentional performance of dyslexics was significantly impaired compared to that of the non-dyslexic group of children. Our group [13] also reported that children with dyslexia were significantly more unstable than non-dyslexic children when reading text silently. Taken together, all these findings suggest that the cerebellum, which is responsible for the integration of proprioceptive inputs during balance, could be impaired in the dyslexic population. It is worth recalling that, similarly to dyslexic children, children with cerebellar deficits have been reported to have poor postural stability, suggesting a difficulty of these patients to integrate multimodal sensory information to control balance [14].

In the present study we aimed to explore further postural control in dyslexic children while performing a dual task. The dual task chosen was a modified Stroop test similar to that used by Olivier et al. [15]. These authors reported a deterioration of postural stability in normal children while performing such a test, most likely because of the increasing attention demanded. It is well known that dyslexic children (7–11 years old) show an important interference effect compared with non-dyslexic children in the Stroop Color-Word Test [16], most likely because of their reduced reading automaticity. In order to avoid stressing dyslexic children with this reading task, we decided to use the modified version of this test already employed in normal children by Olivier et al. [15], in which the words are replaced with fruits.

Our first prediction was that postural stability in dyslexic children would be poor compared with non-dyslexic, age-matched children; this could be true in the baseline condition as well as during dual task in which the fruits were drawn in their natural ripe colour. On the other hand, when the fruits were drawn in an abnormal colour we made the hypothesis that dyslexic children would show even more instability because of the difficulty to share attention between the two tasks and due to the reduced mental flexibility of dyslexic populations according to previous findings [17].

## Materials and Methods

### Subjects

Twenty-one dyslexic children participated in the study. Dyslexic children were recruited from a pediatric hospital where they had been referred for a complete evaluation of their dyslexia with an extensive examination including neurological/psychological and phonological capabilities. For each child, the time required to read a passage of text, text comprehension, and the ability to read words and pseudowords were evaluated using the L2MA battery [18]. This is the standard test developed by the Applied Psychology Centre in Paris (Centre de Psychologie Appliquée de Paris), and used throughout France. Inclusion criteria were scores on the L2MA which were more than two standard deviations from the mean, and a normal mean intelligence quotient (IQ, evaluated using the WISC-IV), namely between 80 and 115 [19], [20]. Mean age of dyslexic children was 10.4±0.3 years, mean IQ was 100±12, and mean reading age was 8.5±0.8 years. Dyslexic children had no sign of hyperactivity or developmental coordination disorder (DCD). We used the Diagnostic and Statistical Manual of Mental Disorders Fourth Edition (DSM-IV), particularly the ADHD Rating Scale to exclude hyperactive children [6]. The ADHD-RS questionnaire was done and for all children its score was lower than 28 [21].

A selected age-matched control group (mean age: 10.5±0.3 years) of twenty non-dyslexic children was chosen. These children had to satisfy the following criteria: no known neurological or psychiatric abnormalities, no history of reading difficulties, and no visual stress or difficulties with near vision. IQ and reading measurements were not available for these children, but their scores for French (reading, comprehension and spelling), mathematics and foreign languages were all beyond the mean scores in their respective school grades. Recruitment of controls based on school performance alone has been used by other researchers [22], [23].

Both dyslexic and non-dyslexic children underwent an ophthalmological examination accompanied by orthoptic evaluation of their visual functions (mean values shown in Table 1).

**Table 1:** Clinical characteristics of dyslexic and non-dyslexic children.

	TNO s of arc	NPC (cm)	Heterophoria (pD)	Divergence (pD)	Convergence (pD)
**Dyslexic children**	54±7	3.7±0.4	−2.2±0.8	11±0.9	29±2
**Non-Dyslexic children**	58±5	3±0.4	−2.6±0.8	18±0.4*	40±2*

Mean and standard deviation values for binocular vision (stereoacuity test, TNO measured in seconds of arc); near point of convergence (NPC measured in cm); heterophoria at near distance measured in prism diopters; fusional vergence amplitudes (divergence and convergence) at near distance measured in prism diopters. Asterisks indicate that the value is significantly different with respect to the dyslexic children group (p<0.01).

Visual acuity was normal (≥20/20) for all children in both groups (see Table 1 for details). All children had normal binocular vision, as evaluated with the TNO Random-Dot Stereotest (Test of Netherlands Organization; Richmond Products, Boca Raton, FL). ANOVA showed a significant difference of the near point of convergence for both groups of children tested (≤7 cm). In addition, an orthoptic evaluation of vergence fusion capability using prisms was carried out at near distances. The phoria (i.e., latent deviation of one eye when the other eye is covered, using the cover-uncover test) was within the normal range for all children tested (mean value: –2.2±0.8 pD and −2.6±0.8 pD in dyslexic and non dyslexic children respectively). The divergence and convergence amplitudes were significantly smaller in the dyslexic group than in the non-dyslexic children. An ANOVA showed a significant main effect of group (F_(1,39)_ = 38.81, p<0.0001 and F_(1,39)_ = 15.49, p<0.0004, for divergence and convergence amplitude, respectively).

By and large, the orthoptic evaluation showed poor vergence fusional capabilities in dyslexic children in line with other studies on this type of children [24], [25].

The investigation adhered to the principles of the Declaration of Helsinki and was approved by our Institutional Human Experimentation Committee (CPP Ile de France I, Hôpital Hotel-Dieu). Written consent was obtained from the children’s parents after an explanation of the experimental procedure.

### Platform

A platform (principle of strain gauge) consisting of two dynamometric clogs (standards of Association Française de Posturologie, produced by TechnoConcept, Céreste, France) was used to measure postural stability. The position of the feet was as follows: heels 4 cm apart and the feet spread out symmetrically at a 30° angle with respect to the child’s sagittal axis. Arms were vertical along the body. The excursions of the center of pressure (CoP) were measured for 26 seconds and the surface of the CoP was calculated following the standards proposed by Gagey et al. [26]; the equipment included a 16-bit analog-digital converter. The sampling frequency of the CoP was 40 Hz.

### Stimuli

Visual stimuli were presented on a flat screen (1280×768 pixels), placed 40 cm away from the children. The elevation of the screen was adjusted as a function of the height of each child so that its center was facing the eyes exactly. In order to avoid giving a reading task to dyslexic children a modified Stroop test was used, in which the words were replaced by fruits. Such methodology is similar to that used by Olivier et al. [15]. The modified Stroop task required the child to name the colour of a fruit (strawberry, banana, apple or orange) appearing consecutively for two seconds on a white screen in front of him/her. Two different conditions were used: (i) a congruent colour condition (C) in which the fruit was drawn in it natural, ripe colour and (ii) a non-congruent colour condition (NC) in which the fruit was drawn in three abnormal colours. In each series, presentation of the four fruits was equiprobable, children had to name the colour of the fruit they were looking at. Note that even if children responded verbally, which is known to affect postural sway [27] and respiratory activity [28], these two factors presumably had similar effects in both cognitive conditions and on both groups of children. Furthermore, a baseline control condition was also recorded in which the child had to fixate a picture of a green apple. The three tasks (two trials per task) were randomly presented. The dimension of the fruit presented on the screen was 2 cm.

### Procedure

Child stood on the platform, both eyes open, in front of the screen. For each task (C, NC and fixation) two postural recordings were taken successively. The order of the visual tasks varied randomly across children. Children were asked to stay as stable as possible, with the arms along the body and to perform the tasks.

### Data Processing

To quantify the effect of tasks and the postural performance from data obtained from the platform, we analyzed the surface area, the length and the mean speed of the center of pressure (CoP). The surface area and the length allowed for the efficient measurement of CoP spatial variability [29]. The surface of CoP corresponds to an ellipse with 90% of CoP excursions. The length of CoP is the path of the center of pressure. Although these two postural parameters are uncorrelated - indeed the inner surface of the same length may be different, they constitute a good index of the amount of neuromuscular activity required to regulate postural control [30], [31]. For each child, we also calculated the number of correct responses in the congruent and non-congruent colour tasks.

### Statistical Analysis

Statistical analysis was performed by the two-way ANOVAs using the two groups of children (dyslexics and non-dyslexics) as inter-subject factor and the three conditions (fixation -Fix-, congruent colour -C- and non congruent colour -NC-) as within-subject factor. Post-hoc comparisons were made with the least significant difference (LSD) test. The effect of a factor was considered significant when the p-value was below 0.05.

## Results

**Figure 1:**
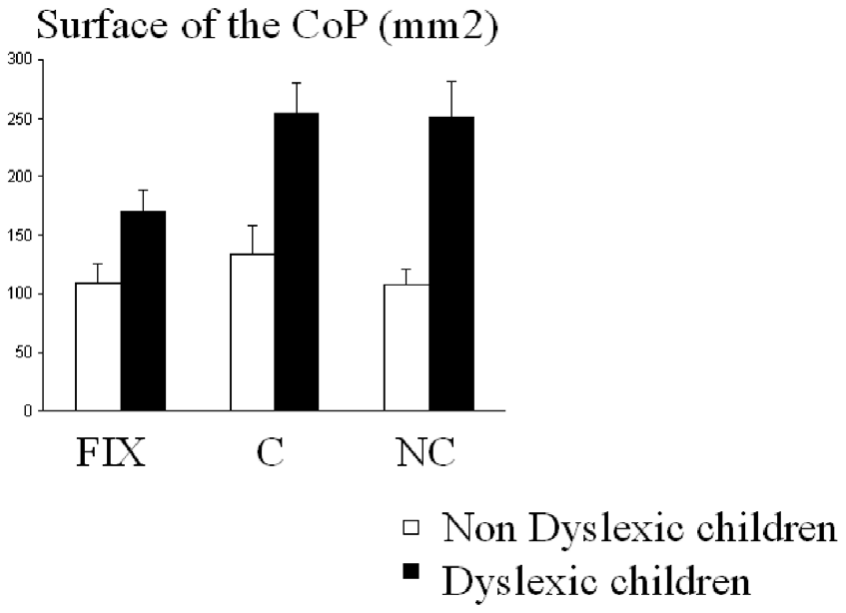
Means and standard deviations of surface area of CoP in mm^2^ in the three conditions (fixation -Fix-, congruent -C- and non congruent -NC-) for the two groups of children (dyslexic and non-dyslexic).

Figure 1 shows the surface area of the CoP for both groups of children during fixation and during the Stroop-like task (congruent and non-congruent trial). Anova showed a significant group effect (F_(1,39)_ = 17.84, p<0.0001): the surface area of the CoP was significantly larger in dyslexic than in non dyslexic children (mean 225±27 mm^2^ and 116±8 mm^2^, respectively). There was also a significant effect of task (F_(2,78)_ = 5.54, p<0.005). Post hoc comparisons showed that the surface area of the CoP during fixation was significant smaller to those reported during the congruent (p<0.001) and the non congruent trial (p<0.02). Interestingly, there was a significant interaction between groups of children and tasks (F_(2,78)_ = 3.14, p<0.04); post-hoc comparisons showed that for dyslexic children during fixation the surface area of the CoP was significantly smaller than during the Stroop-like tasks, congruent (p<0.0001) as well as non congruent trial (p<0.001).

**Figure 2:**
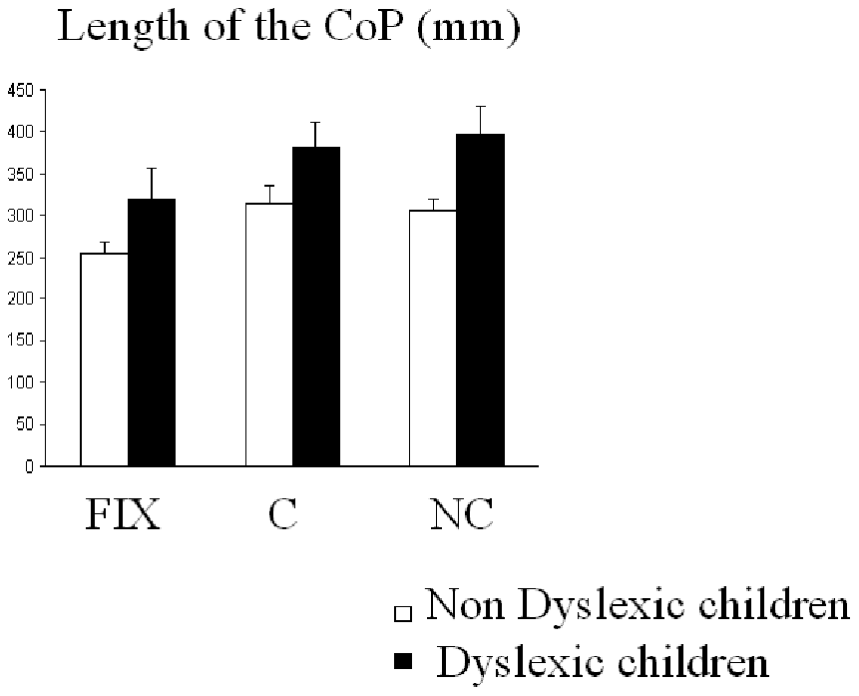
Means and standard deviations of length of CoP in mm in the three conditions (fixation -Fix-, congruent -C- and non congruent -NC-) for the two groups of children (dyslexic and non-dyslexic).

Concerning the length of the CoP (Figure 2) Anova showed a significant effect of the group (F_(1,39)_ = 4.80, p<0.03); the length of the CoP was longer in dyslexic children than in non dyslexic chidlren (mean 367±23 mm and 291±18 mm, respectively). There was also a significant effect of the task (F_(2,39)_ = 15.39, p<0.0001); post-hoc comparisons showed that during fixation the length of the CoP was significantly smaller with respect to congruent and non congruent Stroop-like task (all p<0.0001).

**Figure 3:**
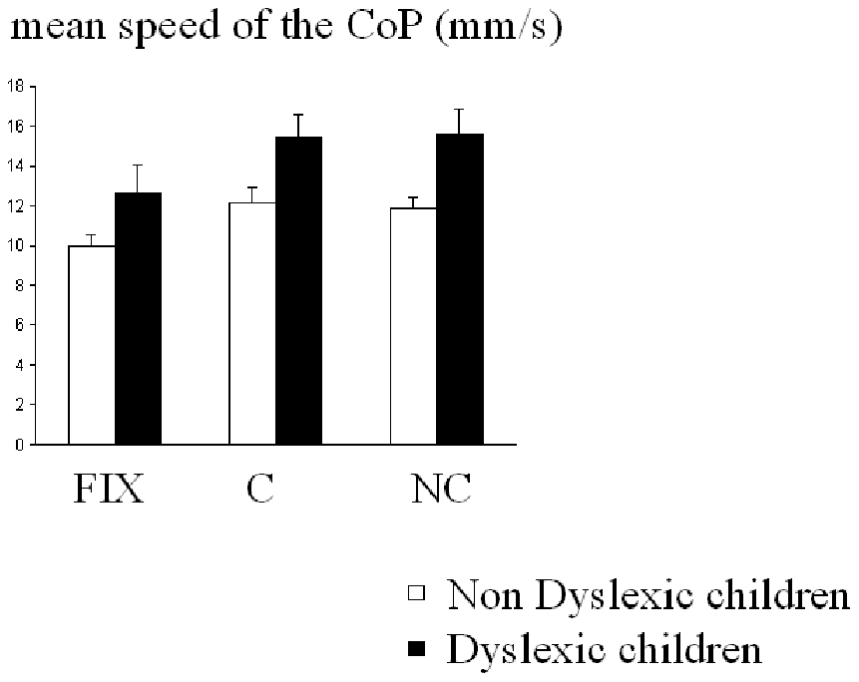
Means and standard deviations of mean speed of CoP in mm/s in the three conditions (fixation -Fix-, congruent -C- and non congruent -NC-) for the two groups of children (dyslexic and non-dyslexic).

The Figure 3 shows data obtained concerning the mean speed of the CoP. Anova showed a significant effect of the group (F_(1,39)_ = 6.13, p<0.01): the mean speed of the CoP was significantly larger in dyslexic than in non-dyslexic children (mean 14±0.9 mm/s and 11±0.7 mm/s, respectively). Anova showed also a significant effect of the task (F_(2,78)_ = 17.35, p<0.0001). Post hoc comparisons reported that during fixation the mean speed of the CoP was significantly smaller than during both and non congruent Stroop-like task (both p>0.0001).

Finally, for both groups of children we evaluated also the number of correct responses during the Stroop-like tasks while performing postural measures. Anova showed a significant difference between the two groups of children (F_(1,39)_ = 20.62, p<0.0001); the dyslexic children had a higher number of errors (wrong name of the colour of the fruit they were looking at) than the non dyslexic children (76±15 and 49±18, respectively). Anova showed also a significant effect of the task (F_(1,39)_ = 28.10, p<0.0001): for both groups of children the number of errors was significantly higher in the NC condition.

## Discussion

The main findings of this study are as follows: (1) postural stability is poor in dyslexic children compared with age-matched non-dyslexic ones; (2) the dual task affects postural control: both groups of children, dyslexic and non dyslexic, decrease their postural stability while performing a secondary Stroop-like task. These findings will be discussed individually below.

### Poor Postural Control in Dyslexic Children

This study shows that postural control in 10-year-old dyslexic children is poor. Indeed all postural parameters measured (surface, length and mean speed of the CoP) were significantly greater in dyslexic children with respect to age-matched non-dyslexic children. This impairment in postural control occurred while children fixated a target as well as when they were asked to make a cognitively demanding secondary task. The effect of the dual task on posture will be discussed in the next sub-section.

The present results confirm and expand previous findings reported in the dyslexic population showing poor postural control in dyslexic children firstly suggested qualitatively by Levinson [7], who advanced the hypothesis of cerebellar impairment in dyslexia. Rae et al. [32] found biochemical differences between dyslexic adults and controls in the left temporo-parietal lob and right cerebellum. These authors found lateral biochemical differences in dyslexic adults in both these brain regions while for controls such lateral difference has not been observed. Eckert et al. [33] using a MRI scan found that children with dyslexia had smaller right anterior lobes of the cerebellum, pars triangularis bilaterally, and cerebral volume. Measurements of the right anterior lobe of the cerebellum and bilateral pars triangularis classified 72% of children with dyslexia and 88% of controls correctly. These measurements also were correlated with reading, spelling and language ability.

This hypothesis has been confirmed later with behavioral studies conducted by several researchers (see also Introduction). For instance Stoodley et al. [34] found that the balancing ability (standing up on the left or right foot) of dyslexic children was significantly less than that of control children. These authors suggested that cerebellum deficiency and the magnocellular immaturity could be at the origin of such impaired balancing in dyslexia. In line with the cerebellar deficiency hypothesis, Barela et al. [35] have advanced the idea that the automaticity responsible for coordinating sensory and motor information in order to obtain adequate postural control could be impaired in dyslexic children.

Postural control involves a complex relationship between sensory and motor information [1]. All these processes seems to be accomplished without considerable cognitive effort [36], [37] at least in normal children, but not in dyslexic children given their limited ability to automatically couple sensory information and motor activity to achieve a correct postural position. Indeed, the same group of researchers [38] have shown that dyslexic children have a deficit in correctly associating visual/sensory information and motor response, suggesting that automaticity is impaired in dyslexic children. Such poor automaticity in children with dyslexia could be the cause of poor ability in reading, writing, and performance of other tasks such as postural control.

Finally, one might ask whether dyslexic children could be trained to develop such automaticity. Indeed cerebellar disorders such as poor control of both stance and gait could be overridden by efficient rehabilitation [39]. Consequently, specific training programs could be developed in order to improve postural control in dyslexic populations. From our point of view, this is the major goal that researchers and therapists need to achieve in collaboration and further studies on such issue need to be done in order to show that improving balance improves reading capabilities in dyslexic population.

### Stroop-like Task affects Posture

This study confirms the previous work done by Olivier et al. [15] in which the same test had been used in normal children but different postural parameters had been measured (the mean speed and standard deviation of the antero-posterio displacement of the CoP for Olivier’s study versus the surface, length and mean speed of the CoP for the present study). According to Olivier’s study, the congruent and non-congruent condition of the Stroop-like task affects postural control in 10-year-old children in a similar way. The new contribution of our study is that such a task has the same effect on posture for dyslexic children in spite of their poor postural capabilities. It should be noted that even if the two conditions has a similar effect on posture, the non-congruent condition is a more difficult task for both groups of children (dyslexic and non-dyslexic); indeed they significantly make more errors in this task than in the congruent one. For dyslexics, the errors done in the non-congruent task was higher; this could be due to their difficulty in allocating attention correctly, as attentional deficits in children with dyslexia have been already reported [20].

On the other hand, we have to point out that even if attentional load significantly increased by incongruency, postural instability is similar in the two conditions (congruent and non-congruent one). This finding could be explained by the hypothesis that the dual-task attentional demand was based on a multiple attentional model with two independent attentional mechanisms [5], [15]. A first attentional mechanism would be involved to the control of posture and a second attentional mechanism dedicated to the control of the secondary task. These two independent mechanisms may interfere when the capacity of one of them is exceeded. The increased complexity of the secondary task (non-congruent condition) could lead to interaction between these two attentional mechanisms. Other studies dealing with attentional demand in dual-task situation are needed to known further how children control postural attentional mechanism.

Taken together, the results on dyslexic as well as on non-dyslexic children are in line with the cross domain competition model described by Lacour et al. [40]. Examining the effect of a secondary task on postural stability, the authors observed that a secondary task affects postural stability; because of attentional resource sharing, balance performance should be less efficient in dual-task conditions. In our study, in the absence of a secondary task (e.g. in the simple fixation of a fruit), the child’ attention is directed to postural control thereby improving stability; in contrast a more complex cognitive task (as Stroop-like task) could be responsible for shifting the attention away from postural control, decreasing postural performance.

Finally, it should be mentioned that the sharing of attention is particularly relevant for children’s daily activities because they frequently encounter situations involving the performance of two tasks at a time. Dual task conditions could be difficult for children, particularly dyslexic populations, for whom focusing attention is difficult. Furthermore, therapists may need to adapt their clinical examinations to children showing difficulty under a dual task condition (as dyslexic population): providing a quiet environment with limited concurrent tasks could be more conducive to accurate clinical assessment and possible intervention.

### Conclusion

In 10-year-old children (dyslexic and non dyslexic) a secondary task such as a Stroop-like task requiring shifting attention for executing this task correctly leads to a decrease in postural stability.
