# Correction: The Effect of a Stroop-like Task on Postural Control in Dyslexic Children

**DOI:** 10.1371/annotation/7823a3d4-5d4c-4b4b-b6c2-aa9573051565

**Published:** 2014-01-14

**Authors:** Maria Pia Bucci, Emmanuel Bui-Quoc, Christophe-Loic Gerard

There is an error in the first author's name in the citation. The correct citation is:

Bucci MP, Bui-Quoc E, Gerard C-L (2013) The Effect of a Stroop-like Task on Postural Control in Dyslexic Children. PLoS ONE 8(10): e77920. doi:10.1371/journal.pone.0077920

